# Sarcoidosis and Its Dermatological Manifestations: A Narrative Review

**DOI:** 10.7759/cureus.28053

**Published:** 2022-08-16

**Authors:** Jancy Koneti, Swathi Priya Cherukuri, Sai Gadde, Revanth Kalluru, Rahul Chikatimalla, Thejaswi Dasaradhan

**Affiliations:** 1 Research, Kamineni Institute of Medical Sciences, Narketpally, IND; 2 Research, Narayana Medical College, Nellore, IND

**Keywords:** subcutaneous nodules, erythema nodosum, lupus pernio, cutaneous sarcoidosis, sarcoidosis

## Abstract

Sarcoidosis is an enigma diagnosed by ruling out other etiologies of granulomatous inflammation. The multisystem manifestations of sarcoidosis and the clinical polymorphism pose a diagnostic challenge to all physicians. The skin is the most commonly affected organ after the lungs in sarcoidosis. Dermatological manifestations can appear before, during, or after systemic involvement, and the type of skin lesion can have prognostic significance. Also, a biopsy of skin lesions is less invasive and more accessible to perform than a biopsy of visceral organs. Thus, in certain ways, cutaneous manifestations can aid in the diagnosis and prognosis of systemic disease. This article has focused on the frequently encountered skin lesions of sarcoidosis along with their prevalence, clinical features, and management.

## Introduction and background

Sarcoidosis is a multisystemic disease that affects people from all over the world. It is characterized by the presence of non-caseating granulomas in the involved organs. Granulomas can be found in any organ, but the lungs are the most common organs involved with bilateral hilar lymphadenopathy [1].

Jonathan Hutchinson described the first case of sarcoidosis in 1875 when it was more of a dermatological curiosity. However, as science progressed and more research was conducted, the disease gradually evolved into a multisystemic one [2,3]. Globally, the incidence of sarcoidosis is estimated to be roughly 16.5 per 100,000 men and 19 per 100,000 women [4]. Women (1.3%) had a greater lifetime incidence than men (1%), and African Americans (2.4%) had a higher lifetime incidence than Caucasians (0.8%) [5,6]. People of any age are susceptible to the disease, but a tendency is seen in both genders between the ages of 25 and 40 in Scandinavian nations and Japan, the second peak in occurrence has been documented in women over 50 in some but not all published studies [7]. An environmental insult, such as exposure to infection, toxins, and heavy fumes, most likely causes sarcoidosis in a genetically vulnerable host. The high incidence and prevalence of sarcoidosis in African Americans suggest a genetic predisposition. First-degree relatives of African Americans with sarcoidosis have a threefold increase in disease risk [8]. Candidate gene and genome-wide association studies have looked into many sarcoidosis susceptibility loci. Among them are the human leukocyte antigen (HLA) genes, which are found in class II major histocompatibility complex (MHC) on chromosome 6, and other genes that influence antigen processing and presentation, T-cell activation and recruitment, and granulomatous inflammation at the core of sarcoidosis immuno-pathogenesis [9].

Sarcoidosis has a variety of clinical symptoms, but virtually all patients experience respiratory tract involvement at some point. The most common symptoms are dry cough, dyspnea, and chest discomfort, particularly in patients with severe endobronchial or parenchymal involvement [10]. The next most prevalent illness sites are the skin, eyes, liver, spleen, and peripheral lymph nodes, with involvement rates ranging from 10% to 30% [11]. Cardiac and neurological problems are infrequent but can be fatal [12,13]. The diagnosis is not standardized but based on three critical criteria: a similar clinical or radiological presentation, histological evidence of non-necrotizing granulomatous inflammation in one or more tissues, and the exclusion of other granulomatous disease causes. Certain clinical manifestations like Lofgren's syndrome, lupus pernio (LP), and Heerfordt's syndrome are considered highly specific for the disease and do not require histological confirmation [14]. Although the best treatment for sarcoidosis is unknown, corticosteroid medication has long been the standard of care for individuals with severely symptomatic or advancing pulmonary disease or substantial extrapulmonary illness. Immunomodulators may be required in complex instances [15]. Despite its nineteenth-century origin and more than a century of research, much about sarcoidosis remains unknown. Perhaps this is due to its numerous points of divergence - various epidemiologies, varying triggering exposures, and, ultimately, variable clinical phenotypes [16].

The underlying mechanism of sarcoidosis is believed to be a link between genetic susceptibility and environmental or infectious factors. Additional research is needed to determine the exact cause of sarcoidosis, how granulomas form, and the various skin lesions that sarcoidosis can cause such as erythema nodosum and LP, maculopapular, nodular, and annular lesions. Skin sarcoidosis affects roughly 25% of people with systemic illness and may also arise in isolation [17]. A variety of clinical presentations of cutaneous sarcoidosis (CS) is recognized. The degree of systemic involvement determines the therapy and overall prognosis of CS [17]. This article casts light on the diversity of CS presentations, as well as the management of them, as lesions can take on a variety of morphologies, posing a challenge to physicians in diagnosing them.

## Review

**Figure 1:**
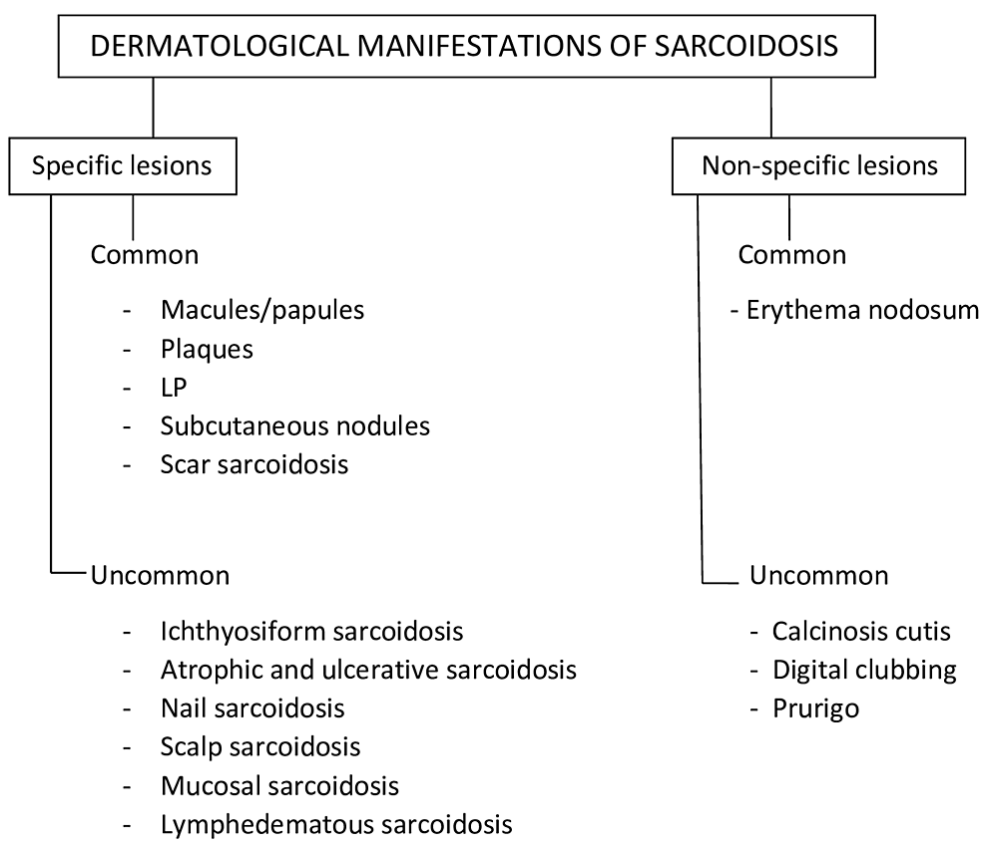
Common and uncommon dermatological manifestations of sarcoidosis LP: lupus pernio Image credits - Jancy Koneti

CS is known as the great imitator because of its wide range of morphologies [18]. It is categorized based on the existence of non-caseating granulomas on biopsy. There are two types of skin manifestations of sarcoidosis: 1. Specific lesions that are defined by the presence of non-caseating granulomas in skin biopsies, such as maculopapules, plaques, subcutaneous nodules, LP, and infiltrative scars, and other uncommon forms such as ichthyosiform sarcoidosis, atrophic and ulcerative sarcoidosis, nail, scalp, and mucosal sarcoidosis; 2. Non-specific lesions. These lesions represent a reactive process to systemic sarcoidosis but lack granulomas on biopsy. Erythema nodosum is the most common non-specific lesion. Other rare forms include calcinosis cutis, digital clubbing, and prurigo (Figure 1) [18,19].

Cutaneous lesions can aid in diagnosing sarcoidosis and are easily accessible tissues for biopsy [20]. It is critical to recognize these skin lesions because they allow for early diagnosis of the systemic disease. Some types of lesions have prognostic significance and may help predict the outcome of the systemic disease. Specific skin lesions are linked with worse results and are more challenging to treat than non-specific lesions [19,21]. While sarcoidosis cutaneous lesions rarely cause significant morbidity or mortality, their disfiguring nature can have a significant psychosocial impact. The key issue of sarcoidosis is the difficulty in treating these various skin lesions [19]. Intralesional corticosteroids and high-potency topical corticosteroids have been demonstrated to be effective in managing these lesions [22]. Systemic treatment is usually not indicated unless the lesions are progressive and disfiguring. It is reserved for patients with severe or progressive pulmonary symptoms because many patients have spontaneous remissions, and the benefits of therapy do not affect the long-term outcome [23].

Common dermatological manifestations of sarcoidosis will be discussed in the subsequent text.

Papular and plaque sarcoidosis

The most frequently seen variants of CS are papules and plaques. As per a study by Rim Ishak et al. in 2015 on 76 patients from the American University of Beirut Medical Center diagnosed with CS between 1992 and 2010, 37 patients had plaques (49%) and 22 patients had papules (29%) [24]. The findings of the above study can be compared along the lines of another study that was conducted by Lidia García-Colmenero et al. in Spain in 2019, in which 40 patients with cutaneous manifestations of sarcoidosis were evaluated, out of which 20 patients had papules and 12 patients had plaques, concluding that papules and plaques are the most common CS variants [25].

Maculopapules are red-brown to purple, less than 1 cm in diameter, and very weakly infiltrated with some epidermal alteration. They are less commonly skin-colored, yellow-brown, or hypopigmented. The papules have a characteristic translucent yellow-brown tint in the background of erythema. The distinctive apple jelly color of the granulomatous skin lesion is seen on diascopy [19,26]. An umbilicated center can be found on some of them. These papules occasionally combine to form annular lesions [27].

They're widely diffused and found predominantly on the face and neck, especially on the eyelids, around the orbits, and in the nasolabial folds, as well as the occipital area of the neck [27]. Recent studies suggest that sites of repetitive trauma, such as knees, can be a predilection for papular sarcoidosis. According to this study by J Marcoval et al. in Bellvitge University Hospital in 2011 on 86 patients with systemic sarcoidosis and granulomatous cutaneous involvement followed up for more than two years, maculopapules were more commonly located on or around the knees [21]. It is mainly observed in acute forms of sarcoidosis and can be considered a sign of good prognosis [21,28,29]. According to a study performed by J Mañá et al. in 1997 on 37 patients with cutaneous involvement in sarcoidosis, 14 patients had maculopapules (38%), and only four cases among those 14 showed disease activity for more than two years. These lesions often self-resolve without scarring [21]. As the lesions are frequently found on the face, the differential diagnosis includes perioral dermatitis, tinea faciei, cheilitis granulomatosa, cutaneous Crohn's disease, granuloma faciale, lymphocytoma cutis (Lyme borreliosis), lupus vulgaris, lupus miliaris disseminatus faciei, and lupus erythematosus [30].

Papules, notably on the extensor surfaces of arms, face, scalp, back, and buttocks, can progress into plaques greater than 5 mm [27,31]. These lesions have an oval or circular shape, are often clearly defined, are firm to the touch, and can be scaly. Their color ranges from red-brown to flesh-colored to purple-brown to yellow-brown at times [20]. Plaques tend to be thicker, fewer in number, and are indurated and raised in appearance. They reflect a more extensive infiltrate than papules, and noncaseating granulomas can be found throughout the dermis. The typical yellow-brown tint may be revealed during a diascopy. Differentiation of papular and plaque sarcoidosis is salient because of the prognostic relevance. Patients with plaques are more likely to have a chronic disease course [27]. Plaques are more likely to cure with persistent scarring than papular lesions [31]. Other dermatoses with scaling and central atrophy that can be confused with plaque sarcoidosis are chronic discoid lupus erythematosus, granuloma annulare, psoriasis, discoid eczema, syphilis, mycosis fungoides, tinea corporis, leishmaniasis, non-tuberculous mycobacteriosis of the skin, and lichen planus [30].

Subcutaneous sarcoidosis

Subcutaneous sarcoidosis is the least common among the specific lesions of the CS. Its incidence is high in the fourth decade and most common in women than men. Multiple, firm, asymptomatic to slightly painful, mobile, round to oval, skin-colored nodules frequently occur in a bilateral and asymmetric pattern on the extremities [32]. Ahmed et al. conducted a retrospective review study in 2006 in which 21 patients with subcutaneous sarcoidosis were identified between 1996 and 2001 from their institute records. They were 15 women and seven men. The mean age was 46.3 years. This study and a literature review of published 33 cases of subcutaneous sarcoidosis concluded about the lesional multiplicity, the sites of involvement, and association with systematic disease. The number of lesions in each patient was more than six in 18 of 21 patients (86%). They were asymptomatic in 12 patients while the remaining nine had mildly tender lesions. Subcutaneous lesions were located on the upper extremities in all 21 patients while 16 patients had them on the lower extremities. Other body parts that can be affected include the trunk, face, buttocks, head, and neck [32]. As it is mostly asymptomatic, it may go unnoticed and underreported by non-dermatologists [33].

Subcutaneous sarcoidosis typically manifests at the start of the disease, and it usually indicates sarcoidosis with non-severe systemic involvement and is not linked to chronic fibrotic illness. A clinicopathological study by J Marcoval et al. in 2005 revealed that in 85 of 480 patients with systemic sarcoidosis, granulomatous cutaneous involvement was found. Subcutaneous sarcoidosis was diagnosed in 10 of the 85 cases, and in all these 10 cases, subcutaneous nodules appeared at the beginning stage of the disease. None experienced chronic or severe sarcoidosis consequences such as pulmonary fibrosis and bone cysts [34]. Differential diagnoses of subcutaneous sarcoidosis include rheumatoid nodules and xanthomas [30].

The need for and the type of treatment depends on the severity of the systemic condition. Systemic corticosteroid therapy is the basis of treatment for patients with significant systemic involvement or disfiguring skin lesions. The therapeutic response of skin lesions to oral corticosteroids was observed in all patients who received corticosteroids as their primary treatment in a series conducted by Ahmed and his colleagues as mentioned above. The daily dosage ranged from 20 to 40 mg, and responses were frequently evident four to eight weeks after treatment began [32]. In rare instances, doxycycline has been effective in treating subcutaneous sarcoidosis rather than corticosteroids. A case report published by Fouad El Sayed et al. on a 45-year-old lady with subcutaneous nodules for seven years and new onset of dyspnea showed that complete remission was achieved after a six-month course of doxycycline at a dose of 200 mg/dl. She was also treated with corticosteroids, but her disease resurfaced upon withdrawal [35].

Scar sarcoidosis

Lesions of CS can also appear in pre-existing scars [18]. A scar can be caused by an injury, surgery, a burn wound, or a tattoo [36]. The damaged skin sites serve as the foundation for the new concept of an immunocompromised cutaneous district (ICD), which refers to a skin area with acquired immune dysregulation that can lead to the local onset of opportunistic disorders such as infections, tumors, and granulomatous disorders [37]. It is a common variant of CS. In a clinical study by Neville and his colleagues in 1983, 29% of patients with CS had scar sarcoidosis [38].

Clinically, the lesions present as papules, plaques, nodules, and Ill-defined erythemas. Plaque formation was seen in the scars of eight patients in a study by T Atci and his colleagues [36]. When non-caseating granulomas infiltrate scars, previously quiescent scars turn into purple and tender lesions [27]. Sarcoidal granulomas can also infiltrate old tattoos and places with foreign-body material [29]. It can also develop at the sites of other injections such as silicone and desensitizing injections [39,40]. Scar sarcoidosis may occur after erythema nodosum in the acute phase of the disease. In contrast, it is associated with long-term pulmonary and mediastinal involvement, uveitis, peripheral lymphadenopathy, bone cysts, or parotid enlargement in chronic sarcoidosis [41,42].

Examining the scars of individuals with suspected or confirmed sarcoidosis is significant since a biopsy of these scars can be easily performed, confirming the diagnosis and preventing more invasive examinations [23]. Keloids or hypertrophic scars should be ruled out before diagnosing scar sarcoidosis, as they share a resemblance [43]. According to Veien et al., two years following diagnosis, 22 out of 26 (85%) patients with scar sarcoidosis had a chronic active illness. However, scar infiltration was not related to acute or chronic types of systemic sarcoidosis in a recent investigation on the prognosis of CS, implying that it has little prognostic importance [21,22]. Scar sarcoidosis often resolves gradually and spontaneously [23]. Patients with sarcoidosis can be counseled to avoid tattoos to help prevent disease development [44].

Lupus pernio

LP is the most distinctive cutaneous lesion of sarcoidosis. The exact mechanism of development of LP in cases of sarcoidosis is unknown. It is more commonly seen in women with long-standing sarcoidosis, as evident in the subsequent studies discussed below [19,26,45]. A study done by Spiteri in 1985 on 818 patients with clinical and histological evidence of sarcoidosis showed that 35 patients had LP and its prevalence was twice in females. This study revealed that compared to the overall prevalence of sarcoidosis, LP was more prevalent in West Indians than in Caucasians [46]. It presents on the nose, middle face, and cheeks as red to violaceous, indurated plaques with infrequent superficial scaling leaving behind telangiectatic scars, which are very unsightly [26,31]. There is a close association between upper respiratory tract sarcoidosis and LP, and they both are indicators of chronic sarcoidosis [45]. Patients with LP have a high rate of intrathoracic, upper respiratory tract, ophthalmic, bone cysts, and reticuloendothelial involvement. Compared to other types of CS, the frequency of bone involvement, most typically of the fingers and toes, has been observed to be higher in patients with LP. According to the study performed by Spiteri et al. as mentioned above, 46% of patients with LP had bone cysts in both hands and feet [46,47]. Rhinophyma, lupus erythematosus, lymphomas, and cutaneous angiosarcoma are among the differential diagnoses of LP [30].

LP can be a vigorous disease that obstructs the upper respiratory tract internally and externally, necessitating aggressive therapy [27]. It is a disfiguring skin lesion of sarcoidosis that is very difficult to treat and frequently has a significant psychosocial impact on the patient's quality of life [48]. Infliximab regimens were found to be more effective than other agents in attaining resolution or near resolution. A study done by Stagaki et al. in the medical university of South Carolina clinic in 2008, which described 54 patients with sarcoidosis with LP, who underwent 116 different treatments over eight years, showed that for the treatment of LP, infliximab appears superior to systemic corticosteroids with or without additional agents [48]. Another study published by Yanardag in 2003 contrasted the above research. Of 513 patients with sarcoidosis, 14 were diagnosed with LP, and most recovered by using steroids. However, they also mentioned the need for exploring new treatment modalities for sarcoidosis patients with exclusive skin involvement [49]. Despite its striking appearance, LP appears uncommon among the dermatological manifestations of sarcoidosis.

Erythema nodosum

It is the most common non-specific cutaneous lesion of sarcoidosis as no granulomas are found on histological examination. Approximately 10-22% of all erythema nodosum cases are caused by sarcoidosis [50]. Its most commonly seen in women and people of Northern European descent [10]. A review published by James and Williams found 286 cases, 11% of which were specific skin lesions and 17% of which were erythema nodosum. Lesions were more common in women than in men (2:1) and were more common in blacks than in other races [51].

It is an acute marker of sarcoidosis and is believed to be triggered by the undergoing inflammatory process. Inflammation of the subcutaneous fat gives rise to tender, warm subcutaneous nodules that range in color from erythematous to violaceous to brown. It is most commonly observed on the shins [20]. There are several causes for erythema nodosum, but when there is bilateral hilar lymphadenopathy, it is most likely attributable to sarcoidosis. In a study conducted by James et al. in 1956 on 27 patients with erythema nodosum due to sarcoidosis, chest radiographs of 24 patients showed bilateral hilar lymphadenopathy [51]. It's frequently linked to arthralgias, periarthritis, edema in the lower extremities, and fever. According to the study mentioned above, conducted by James on 27 patients, erythema nodosum was preceded or followed by transient polyarthritis in 17 cases [51].

Triad of erythema nodosum, bilateral hilar lymphadenopathy on X-ray, and joint symptoms such as arthritis of the ankles, wrists, and elbows make up Lofgren's syndrome, which is highly specific for sarcoidosis [52]. Though the initial phase can be pretty severe, Lofgren's syndrome has a favorable prognosis and spontaneous remission [31,51]. Erythema nodosum has been attributed to a better clinical outcome. As per a study conducted by Neville and his colleagues in 1983 on 818 patients diagnosed with sarcoidosis, only 16% of patients with erythema nodosum pursued a chronic course while the remaining patients showed good prognosis [38].

Diagnosis and management

To establish the diagnosis of CS, a biopsy and histopathological examination is required, along with the morphological presentation. Sarcoidosis should be well-considered in the differential diagnosis of various skin lesions [31]. It must be differentiated histopathologically from leprosy and lupus vulgaris, as they all have epithelioid cell granulomas. Unlike granulomas in lupus vulgaris, which are caseous and found in the upper dermis, those in leprosy are found primarily around dermal nerve twigs and are combined with lymphocytic infiltration. Sarcoidal granulomas, on the other hand, are distinct, uniformly distributed in the dermis, surrounded by scanty lymphocyte cuffing (naked tubercles) with fine reticulin fibers in and around the tubercles [41]. When a skin biopsy raises suspicion of sarcoidosis or a diagnosis of sarcoidosis is expected, a systemic assessment is required, which includes a detailed history, physical examination, and laboratory and radiographic evaluation [31]. The Kveim-Siltzbach skin test, which involves injecting spleen or lymph-node homogenate from a sarcoidosis patient intradermally and then performing a biopsy, is not generally available, standardized, or Food and Drug Administration (FDA)-approved for general usage [53].

Treatment options are primarily based on circumstantial evidence. Observation without therapy is recommended, as most lesions are asymptomatic and often resolve spontaneously. However, disfiguring and extensive skin lesions associated with severe systemic illness require treatment [17]. Treatment for CS should be done in stages, considering the risks and benefits of the therapy, with local therapies first (topical and intralesional steroids, tacrolimus, and topical retinoids) and then systemic immunomodulatory treatments (hydroxychloroquine, chloroquine, tetracyclines, thalidomide) alone or in combination, and finally, systemic immunosuppressive drugs such as methotrexate, prednisone, azathioprine and tumor necrosis factor (TNF) inhibitors (adalimumab, infliximab) [20,31].

Small papules and plaques are often treated effectively with monthly intralesional triamcinolone injections. Treatment with a topical corticosteroid and a hydrocolloid occlusive dressing on a weekly basis can also be useful [54]. Clinical improvement was reported in refractory cases of sarcoidosis by using topical tacrolimus twice daily [55,56]. Although antimalarials chloroquine phosphate and hydroxychloroquine are an effective alternative therapy for cutaneous sarcoidosis lesions, the danger of chloroquine-induced retinopathy is alarming. It can be prevented with a carefully calculated daily dosage and regular six-month ophthalmologic follow-up examinations [57,58]. The antibiotics minocycline and doxycycline may help treat CS because of their anti-inflammatory properties. However, randomized controlled trials are necessary to decide the actual efficacy of tetracyclines [59]. In a few cases of resistant CS, thalidomide and tranilast have improved the outcome [60,61]. Treatment with systemic immunosuppressive therapy, such as systemic corticosteroids (prednisone), methotrexate, and azathioprine, should be considered sooner in patients with severe, disfiguring, or resistant illness or in patients with LP [20]. Given the multitudinous side effects caused by systemic corticosteroids, the use of steroid-sparing agents, such as methotrexate and azathioprine, has been advantageous. Symptomatic improvement was noticed with low-dose methotrexate in patients with persistent and symptomatic sarcoidosis, as reported by E E Lower et al. [62]. The therapeutic response, however, could take up to six months. Hematological, gastrointestinal, hepatic, and pulmonary toxicities are among the most common side effects reported with the usage of methotrexate [63]. TNF inhibitors adalimumab and infliximab are effective in treating severe, ulcerative, or refractory sarcoidosis, as well as patients with LP who have not responded to conventional treatments [64,65,66]. Even though several therapeutic options are discussed in the literature, there is no consistently effective treatment for sarcoidosis [67].

Limitations

This review has some limitations. It doesn't include uncommon dermatological manifestations of sarcoidosis like ichthyosiform sarcoidosis, scalp sarcoidosis, atrophic and ulcerative sarcoidosis, mucosal and nail sarcoidosis, and a few of the studies mentioned here are case studies and reports with a very small sample population.

## Conclusions

Sarcoidosis is a multisystem idiopathic disease that forms noncaseating epithelioid cell tubercles in affected tissues or organs. Understanding the wide range of sarcoidosis clinical manifestations can help avoid diagnostic delays. Skin is the first and most common extrapulmonary manifestation of sarcoidosis. The most common cutaneous manifestations are macules, papules, plaques, subcutaneous nodules, LP, scar sarcoidosis, and erythema nodosum. Maculopapules, subcutaneous nodules, and erythema nodosum are typically associated with acute sarcoidosis. They indicate a favorable prognosis, whereas plaque-type lesions and LP are more commonly associated with advanced stages and necessitate treatment. Regardless of the extent of cutaneous involvement, patients with CS must be closely monitored. Sarcoidosis-related skin eruptions are exceedingly diverse and can resemble a variety of skin illnesses. It's tough to classify CS because many kinds appear to be mixed, and there is no consistently effective treatment. More future studies are required on the diagnosis and management aspects to improve the care of patients with CS.
